# Opsoclonus-myoclonus-ataxia syndrome in an elderly female: A video-based case report and literature review

**DOI:** 10.1097/MD.0000000000051387

**Published:** 2026-10-30

**Authors:** Chi Meng, Yan Li, Siyuan Wen, Feifei Chen, Pan Tao, Xin Kang, Jing Huang, Wei Chen, Changqing Zhou

**Affiliations:** a Department of Neurology, Bishan Hospital of Chongqing Medical University, Bishan, China; b Department of Intensive Care Unit, Bishan Hospital, Chonqqing University of Chinese Medicine, Chongqing, China.

**Keywords:** Case report, Dancing Eyes syndrome, Opsoclonus-myoclonus-ataxia syndrome, Parainfectious, Paraneoplastic

## Abstract

**Rationale:**

Opsoclonus-myoclonus-ataxia syndrome (OMAS) is rare in adults. Here, we report a rare case of OMAS in an elderly patient, accompanied by video documentation and a comprehensive literature review, which may offer valuable insights into the clinical features, diagnosis, and management of this condition.

**Patient concerns:**

An 81-year-old female was admitted with limb tremors lasting for 1 day. During the hospitalization, she developed opsoclonus, myoclonus, ataxia, behavioral changes, and sleep disturbances.

**Diagnoses:**

Laboratory and imaging findings were unremarkable, with no tumor detected during follow-up, supporting a diagnosis of idiopathic OMAS.

**Interventions:**

Immunotherapy consisted of steroids, while symptomatic treatment included clonazepam and gabapentin.

**Outcomes:**

The patient showed marked symptomatic improvement after immunotherapy. At two-year follow-up, there was no evidence of recurrence or malignancy.

**Lessons:**

In elderly patients, OMAS is extremely rare and often diagnosed and treated late. Comprehensive screening for malignancies and infectious triggers is critical. Early recognition and prompt immunotherapy improve outcomes.

## 1. Introduction

Opsoclonus-myoclonus-ataxia syndrome (OMAS), also known as opsoclonus-myoclonus syndrome and dancing eyes syndrome, is a rare autoimmune neurologic disorder characterized by opsoclonus (irregular multidirectional eye movements), myoclonus (involuntary muscle jerks), ataxia (impaired coordination or balance), and behavioral changes. Epidemiological data from a retrospective study indicate an annual incidence of 0.27 to 0.40 cases per million in Japanese children.^[1]^ In contrast, adult-onset OMAS demonstrates substantially lower incidence rates, with particularly rare occurrences in octogenarian populations.^[2]^ In adults, OMAS is most frequently attributed to paraneoplastic or parainfectious causes, while autoimmune, toxic, metabolic, and vascular etiologies are rare.^[2]^ Although numerous etiologies have been identified, a considerable proportion of cases remain idiopathic.

Herein, we report a case of OMAS in a previously healthy elderly woman who responded well to glucocorticoid therapy. Additionally, we conducted a comprehensive literature review of this condition to provide a reference for future clinical management.

## 2. Case presentation

An 81-year-old female with no medical history was admitted with limb tremors lasting for 1 day. The emergency brain computed tomography (CT) revealed no significant abnormalities. On admission, she exhibited a respiratory rate of 26 breaths per minute, a heart rate of 70 beats per minute, and a blood pressure of 181/78 mm Hg. She complained of nausea and vomiting. The initial neurological examination revealed unsteady gait and involuntary limb tremors (see Video S1, Supplementary Video, which demonstrates the patient experienced severe myoclonus). Laboratory investigations revealed normal results for complete blood count, complete metabolic panel, thyroid function tests, and C-reactive protein levels. Serological tests for blood-borne pathogens (hepatitis B and C, syphilis, and HIV) returned negative results. A brain magnetic resonance imaging (MRI) scan (Figure S1, Supplemental Digital Content 1) and an electroencephalogram revealed no evidence of cerebrovascular events or epileptiform discharges.

On the third day of admission, the patient’s clinical status deteriorated. Myoclonus showed increased amplitude and duration. Concurrently, she developed multiple neurological deficits, including incomprehensible speech and intermittent confusion. Neurological examination revealed the emergence of bilateral, involuntary, horizontal, and vertical nystagmus suggestive of opsoclonus, which worsened in sustained gaze and persisted during lid closure (see Video S2, Supplementary Video, which demonstrates the patient developed multidirectional opsoclonus). Cerebellar dysfunction progressed, manifesting as impaired coordination in both finger-to-nose and heel-to-knee tests. Given the progressive neurological deterioration, the treatment regimen was escalated with the addition of clonazepam (0.5 mg once daily) to alleviate the movement disorder.

On the fifth day of admission, autoimmune encephalitis and intracranial infection were ruled out by negative antibody test results (NMDA receptor IgG, AMPA receptor 1 IgG, AMPA receptor 2 IgG, LGI1 IgG, CASPR2 IgG, GABAB receptor IgG, IgLON5 IgG, DPPX IgG, GlyR1 IgG, DRD2 IgG, GAD65 IgG, mGluR5 IgG, mGluR1 IgG, Neurexin-3α IgG, DPYSL5 antibody, and Homer3 antibody) and normal cerebrospinal fluid analysis (CSF). The second brain MRI scan again showed no abnormalities (Figure S2, Supplemental Digital Content 1).

On the twelfth day of admission, her condition continued to deteriorate, with progressive cognitive decline and sleep disturbances. Severe ataxia and myoclonic jerks in her trunk and limbs had rendered her unable to sit up in bed or walk. The diagnosis of OMAS was made based on the classic features of opsoclonus, myoclonus, ataxia, and sleep disturbances. Key differential diagnostic considerations in this case were autoimmune encephalitis and intracranial infection. The third brain MRI also showed no abnormalities (Figure S3, Supplemental Digital Content 1). Subsequently, to rule out underlying tumors, we conducted further investigations including serum tumor biomarker levels, paraneoplastic antibody screen (Anti-Hu, Anti-Ri, Anti-CV2, Anti-Amphiphysin, Anti-Ma1, Anti-Ma2, Anti-SOX1, Anti-Tr(DNER), Anti-Zic4, Anti-Titin, Anti-Recoverin, Anti-PKCY, Anti-Yo, Anti-GAD65) in second CSF, serum testing for cerebellar-related antibodies (anti-DPYSL5, anti-Homer3) and thoracic and abdominal CT scans. All findings were negative except for thyroid ultrasound findings of 2 nodules classified as TI-RADS 4a. Given the patient’s financial constraints, dexamethasone (10 mg once daily) was chosen as the preferred therapy rather than intravenous immunoglobulin (IVIG). For symptomatic control, oral gabapentin (0.1 g, tid) and clonazepam (1 mg, qn) were added to address myoclonus and sleep disturbances.

Following 1 week of immunotherapy, the patient demonstrated marked improvement in opsoclonus, myoclonus and ataxia compared to initial presentation. The patient was subsequently discharged with a transition to oral prednisone therapy at 40mg daily. One month after discharge, the patient achieved further recovery of the opsoclonus, myoclonus, ataxia, and behavioral changes, and she was able to walk independently (see Video S3, Supplementary Video, which demonstrates the patient showed further OMAS improvement at 1-month post-discharge). Following two-year follow-up, the patient continued to be clinically stable without signs of neurological symptom recurrence or definitive tumor presence.

## 3. Discussion

OMAS is generally considered a rare autoimmune neurological disorder characterized by opsoclonus, myoclonus, ataxia, and behavioral changes. The variability in clinical presentation and limited awareness among clinicians usually result in misdiagnoses or delayed diagnoses, followed by irreversible neurological damage.^[1]^ Herein, we characterize the etiology, pathophysiology, clinical features, diagnosis, and treatment of adult-onset OMAS, all of which are important for clinicians to achieve early recognition and timely intervention of the challenging clinical syndrome.

The etiology of OMAS varies among adult patients. Paraneoplastic and parainfectious origins constitute a significant portion of the disease. The underlying tumors in OMAS demonstrate significant age-dependent stratification, with neuroblastoma predominating in pediatric populations, while small-cell lung carcinoma and breast carcinoma represent the most frequent malignancies in adults.^[3]^ Adult-onset OMAS may be associated with other malignancies, including ovarian teratoma, ovarian carcinoma, nasopharyngeal tumor, cervical carcinoma, testicular seminoma, etc.^[2,4,5]^ Additionally, OMAS can also occur as a consequence of parainfectious, other autoimmune diseases, toxic, metabolic origins, as well as idiopathic causes.^[2,3]^ Common infectious agents include Epstein-Barr virus, cytomegalovirus, rickettsiae, mycoplasma, herpes simplex virus, varicella-zoster virus, and human immunodeficiency virus.^[2,4,5]^ However, a proportion of idiopathic cases persist in which definitive etiological classification remains elusive despite comprehensive investigations, particularly in older patients.^[2,6]^

At present, the underlying mechanisms of opsoclonus pathophysiology remain unclear, and several theories have been proposed to explain this phenomenon.^[3,5]^ Initially, it was believed that damage to omnipause neurons in the nucleus raphe interpositus of the pons could contribute to opsoclonus. However, there is a lack of neuropathological evidence to support this theory.^[5,7]^ Subsequently, two principal hypotheses were proposed. One is the brainstem theory, which posits that alterations in the membrane properties of brainstem saccadic burst neurons, followed by an increase in neuronal excitability or a reduction in omnipause neuron inhibition, are the basis of opsoclonus.^[8]^ The other is the cerebellar theory, which suggests that disinhibition of the cerebellar fastigial nucleus results from impaired Purkinje cell function, leading to unbridled activation of the omnipause neurons and subsequent saccade generation.^[5,7]^ Among these hypotheses, the cerebellar theory appears to be more persuasive. Evidence from a recent longitudinal fMRI and FDG-PET study demonstrating significant cerebellar fastigial nucleus hypermetabolism provides objective support for the cerebellar theory.^[9]^ Moreover, opsoclonus in adults appears to be associated with decreased cerebellar cortical volume, which supports the cerebellar theory and may explain the correlation between opsoclonus and cerebellar-related symptoms.^[10]^ However, given that saccades are controlled by multiple brain structures, it follows that these theories are not independent but interconnected.

The existing evidence suggests that humoral immunity may play a pivotal role in OMAS pathogenesis. In this process, molecular mimicry between neural antigens and tumor-derived epitopes drives the production of autoantibodies, potentially serving as clinically relevant diagnostic biomarkers for this condition. Several autoantibodies have been identified, such as anti-Ri, anti-Hu, anti-Yo, anti-Ma1, anti-Ma2, anti-CRMP5, anti-GlyR, anti-NMDAR, and anti-DPPX antibodies. However, most OMAS patients test negative for all known antineuronal antibodies.^[3,5]^ Notably, anti-Ri antibodies have been linked to breast cancer, while anti-GlyR antibodies are associated with lung cancer.^[4–6]^ However, the autoantibodies identified in OMAS are not ideal as diagnostic biomarkers due to their limited sensitivity and specificity.^[4,5]^ Further research is required to identify diagnostic biomarkers and elucidate the underlying effector mechanisms.

The typical clinical features of OMAS include opsoclonus, myoclonus, ataxia, sleep disturbance, and behavioral changes. Opsoclonus is a comparatively specific chaotic eye movement consisting of high-frequency, multidirectional, and torsional saccades without an intersaccadic interval. The symptom intensifies with attempts at sustained gaze, persisting during convergence, lid closure, darkness, and sleep.^[3]^ Myoclonus is a sudden, involuntary, and abnormal jerking movement that often involves the trunk, limbs, and head.^[3]^ Ataxia is characterized by gait instability or imbalance, and may arise from myoclonus, cerebellar dysfunction, or a combination of the 2 factors.^[3]^ However, any symptom of the triad may be absent or have a delayed onset in some cases.^[2,3]^ Sleep disturbance and behavioral changes are also frequently observed.^[2]^ It should be noted that a patient’s initial visit may be prompted by some atypical neurological symptoms such as dizziness, balance difficulties, nausea, vomiting, vision abnormalities, tremor, and altered speech.^[2]^ Consequently, early recognition of these atypical symptoms is also crucial because of the rapid progression of the disease.

Assessing the presence of occult malignancy is a critical aspect of the diagnostic workup for OMAS, as the syndrome is typically attributed to the remote effects of paraneoplastic syndromes.^[5]^ For OMAS patients, particularly those aged over 40 years old, the diagnostic workup for an underlying malignancy should include contrast-enhanced CT imaging of the thoraco-abdominopelvic region, brain MRI, and comprehensive serological evaluation.^[3,4]^ Furthermore, positron emission tomography (PET) should be performed when conditions permit. Besides, to reflect neurological inflammation or infection, CSF analysis should include the assessment of antineuronal antibodies, oligoclonal bands, and B-cell activating factor levels.^[5,11]^ Notably, screening for breast and gynecological cancers should be a priority in female patients. Even if all tests are negative, the work-up should be repeated every 3 months as neoplasia can be detected at various stages of paraneoplastic OMAS (P-OMAS).^[3]^

Due to the absence of diagnostic biomarkers, the diagnosis of OMAS is primarily based on clinical presentation and laboratory examination. However, the classic triad of symptoms may not occur simultaneously,^[2]^ which is challenging for early diagnosis. Therefore, the diagnostic criteria for OMAS proposed by an expert panel are recommended.^[12]^ OMAS will be diagnosed if 3 of the following 4 features are satisfied: opsoclonus, myoclonus or ataxia, behavioral changes or sleep disturbance, and tumorous conditions or presence of antineuronal antibodies.

Determining the standardized treatment protocol for adults is a challenge due to the absence of guidelines from large-scale randomized controlled trials. Treatment optimization requires a personalized therapeutic approach tailored to individual patient characteristics, with particular emphasis on targeting the underlying etiology to achieve symptomatic control and disease modification. For infectious etiologies, appropriate antibiotic selection is essential. In P-OMAS, antineoplastic treatment may not achieve complete neurological remission. Nevertheless, tumor resection or targeted therapy should remain the initial step, as these interventions can ameliorate certain neurological symptoms.^[3,6]^

Immunotherapy demonstrates favorable responses across diverse etiologies, representing a promising therapeutic strategy.^[2]^ Immunotherapy options include corticosteroids, adrenocorticotropic hormone (ACTH), IVIG, rituximab, and plasmapheresis, etc.^[2,3,5,13]^ Corticosteroids and ACTH are commonly employed as first-line treatments, and no difference was found between the 2 agents in response to OMAS.^[13]^ Compared to ACTH, corticosteroids offer economic and convenient advantages. IVIG has shown significant therapeutic efficacy in pediatric populations, as evidenced by a phase 3 clinical trial investigating neuroblastoma-associated OMAS.^[14]^ Rituximab is a chimeric monoclonal antibody targeting the CD20 antigen that depletes circulating B cells, which has been widely used for relapsing or refractory cases. Studies have demonstrated that the combination of rituximab with conventional immunosuppressive agents exhibits both therapeutic efficacy and a favorable safety profile as an initial treatment regimen.^[15,16]^ Given that long-term sequelae and OMAS relapses still occur, recent studies indicate that multimodal immunotherapy regimens may improve the prognosis of this challenging disease.^[14,16,17]^ However, the exact choice and duration of immunosuppressive therapy are primarily determined by the severity of the disease and the patient’s tolerance to treatment.

Symptomatic management constitutes an essential component of comprehensive care, particularly for alleviating debilitating neurological manifestations. For patients with prominent opsoclonus accompanied by severe sleep dysfunction, clonazepam represents a first-line pharmacological intervention.^[2]^ Additionally, levetiracetam and gabapentin have demonstrated clinical efficacy in the management of myoclonic symptoms.^[2]^

To our knowledge, adult-onset OMAS is rare, especially in octogenarian populations. Advanced age, severe encephalopathy, and thyroid nodules all pointed towards a P-OMAS diagnosis.^[6]^ However, no definitive evidence of a tumor was identified, as neither fine-needle aspiration biopsy of the thyroid nor PET imaging could be performed due to the patient’s financial constraints, which is regrettable. According to diagnostic criteria, I-OMAS will be diagnosed in OMAS patients without known cancer after a two-year follow-up and no other definitive causes,^[6]^ which allows us to attribute her illness to OMAS associated with idiopathic origin. Although the definitive etiology is unclear, our patient received early immunotherapy with a favorable prognosis, which highlights the importance of timely recognition and early immunotherapy for OMAS patients.

## 4. Conclusion

In conclusion, OMAS represents a rare autoimmune neurological disorder characterized by frequent diagnostic and therapeutic delays, particularly among elderly patients. Early clinical recognition and prompt initiation of immunotherapy are critical for optimizing neurological outcomes. Comprehensive etiological investigation, including thorough screening for potential underlying causes such as neoplastic processes or infectious agents, constitutes an essential component of patient management. For elderly patients presenting with I-OMAS, implementation of long-term follow-up protocols is imperative to monitor for potential tumor development and ensure timely intervention.

## Acknowledgments

The authors thank the patient and her family for consenting to the publication of this work.

## Author contributions

**Conceptualization:** Chi Meng, Yan Li.

**Project administration:** Siyuan Wen, Feifei Chen, Pan Tao.

**Writing – original draft:** Chi Meng, Yan Li.

**Visualization:** Xin Kang, Jing Huang, Wei Chen.

**Supervision:** Changqing Zhou.

**Writing – review & editing:** Changqing Zhou.








