# Supplementary material for: Opsoclonus-myoclonus-ataxia syndrome in an elderly female: A video-based case report and literature review
Source: Medicine (Baltimore). 2026 Oct 30;105(44):e51387. doi: 10.1097/MD.0000000000051387 (PMC13727239; doi:10.1097/MD.0000000000051387)
Supplement: Supplementary file 1 [file medi-105-e51387-s001.pdf]

Supplementary Material: Raw Brain MRI Scans

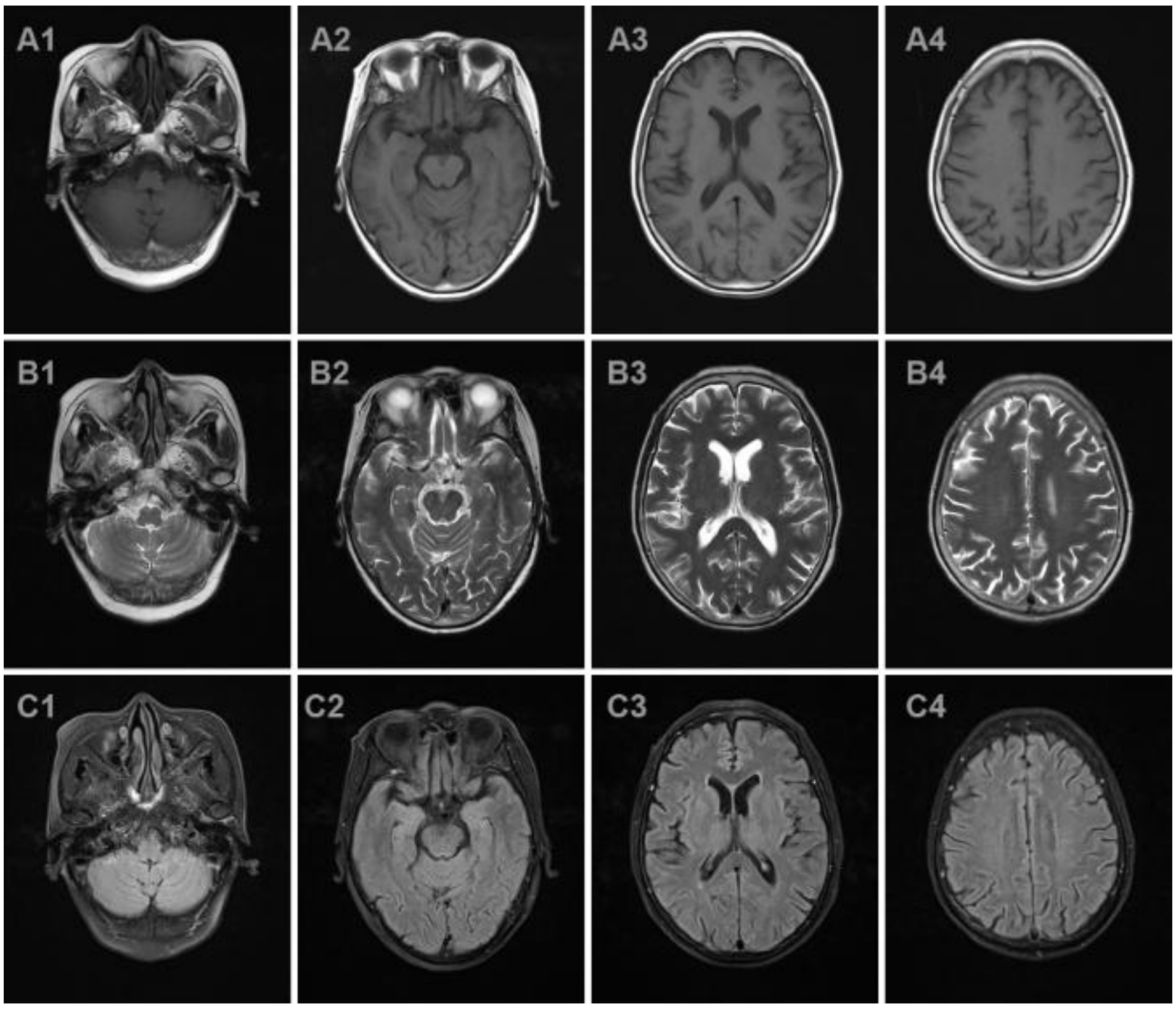

Supplementary Figure 1. The first brain MRI of the patient (On the 2nd day after onset). Axial T1-weighted images (A1-A4); axial T2-weighted images (B1-B4); axial FLAIR images (C1-C4).

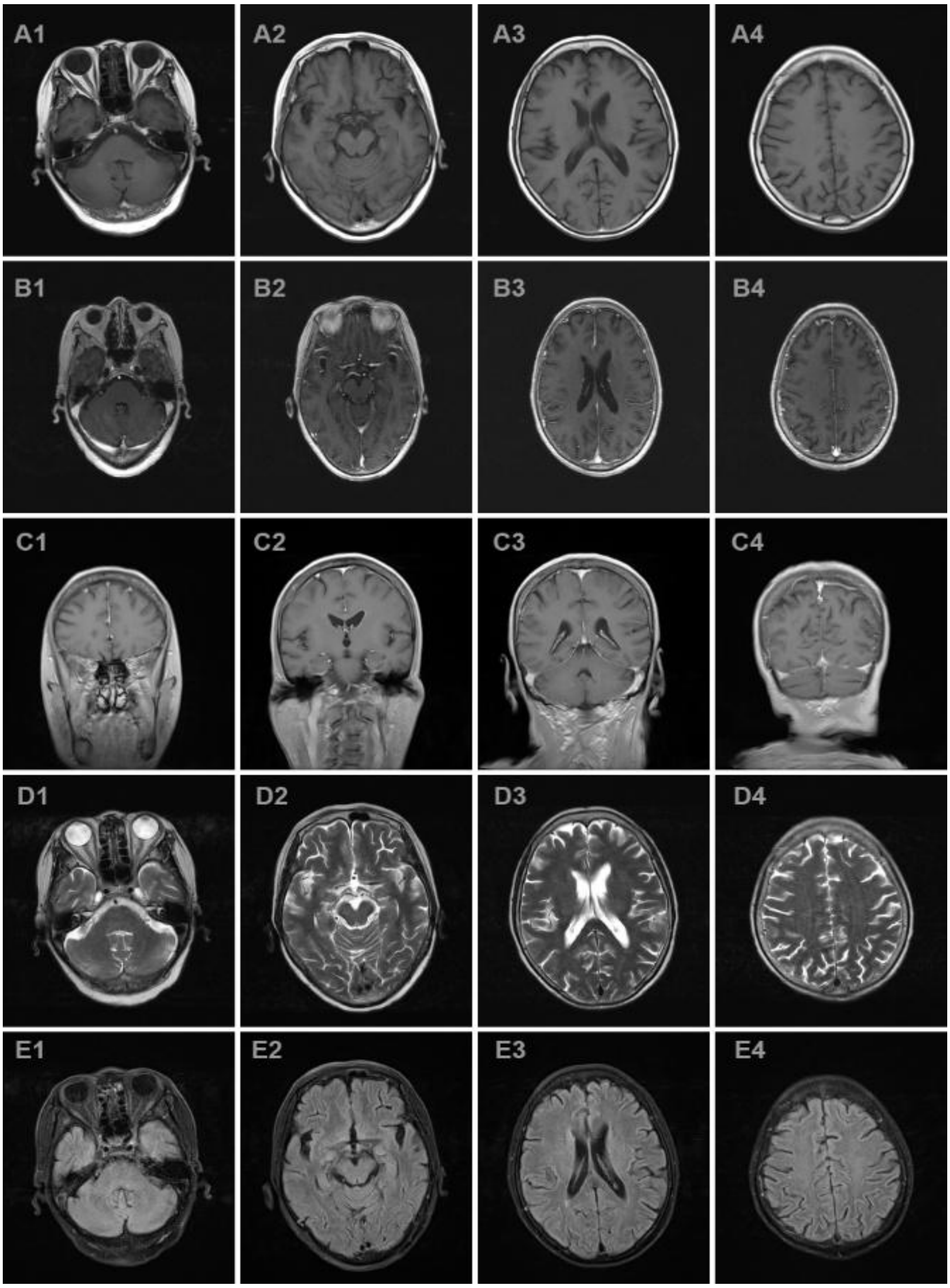

Supplementary Figure 2. The second brain MRI of the patient (On the 5th day after onset). Axial T1-weighted images (A1-A4); axial T1-Gd images (B1-B4); coronal T1-Gd images (C1-C4); axial T2-weighted images (D1-D4); axial FLAIR images (E1-E4).

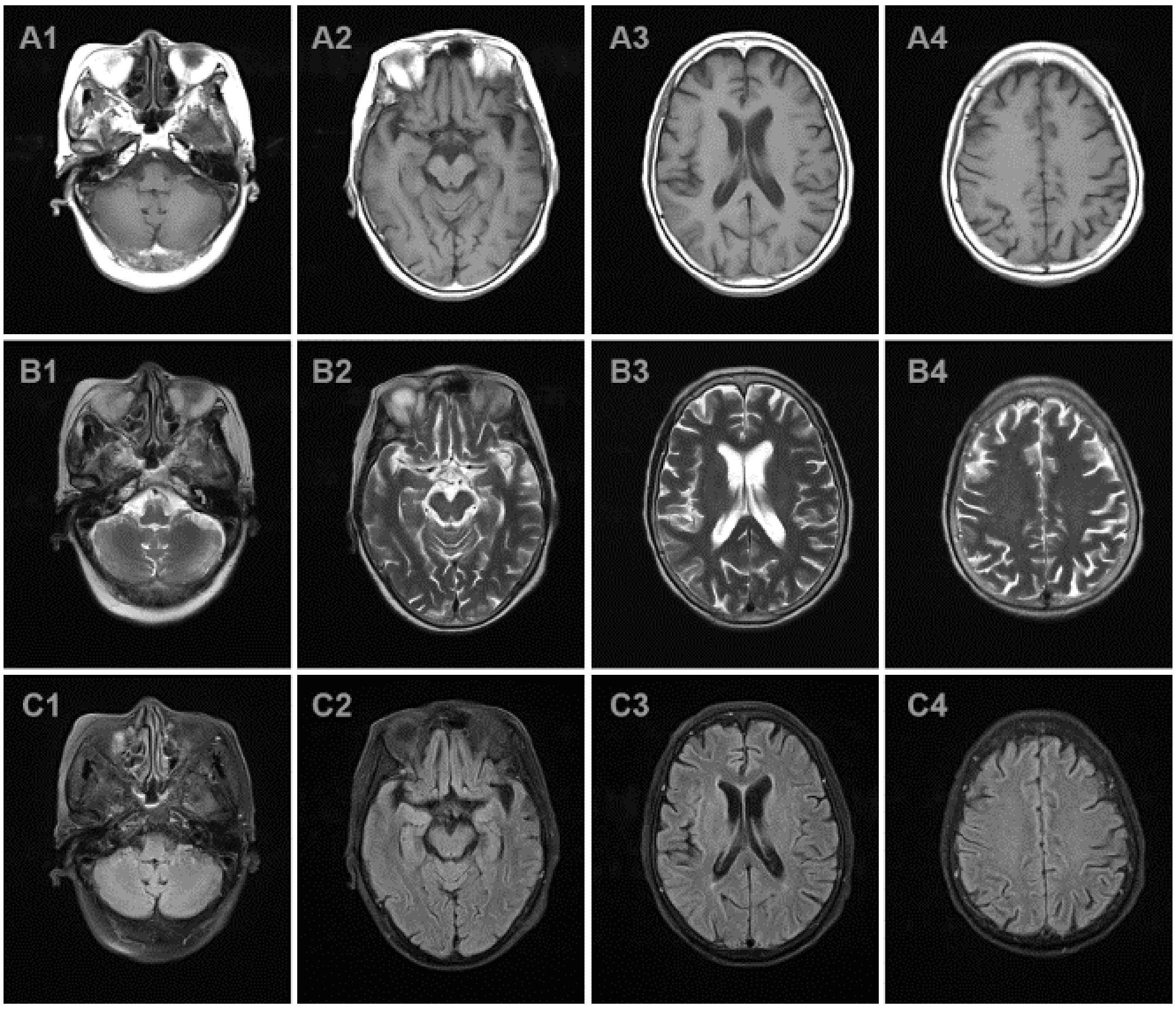

Supplementary Figure 3. The third brain MRI of the patient (On the 13th day after onset). Axial T1-weighted images (A1-A4); axial T2-weighted images (B1-B4); axial FLAIR images (C1-C4).
